# Non-Panel Men and the "Medical Directory"

**Published:** 1914-05-02

**Authors:** F. L. Nicholls

**Affiliations:** Fulbourn, Cambridge


*To the Editor of* The Hospital.

### Non-Panel Men and the "Medical Directory."

*To the Editor of* The Hospital.

Sir,—Your correspondent Dr. A. J. Brock in The Hospital for to-day would appear to have failed to grasp the significance of the term "distinction" used in my letter in a previous issue, and to have failed to see that it merely signified such difference as could easily be adopted by the insertion in brackets of one word after a man's name in the *Directory*.

We must all agree with Dr. Brock that those men who from economic pressure, such as those living in low-class industrial districts where the whole population comes under the Insurance Act, are entitled to our utmost sympathy; but what is to be said of men living in rural districts and good-class towns, who created, in many instances *en masse*, such a spectacle of the profession in the early days of 1913? Are not such worthy of our condemnation and contempt? There will always be touts and black sheep in all professions, but was that any reason why so many members of the profession should have gone on the "panel," as they stated was the cause for their so doing?

Has the profession, as a body, so little belief in the acumen and sound common sense of the public as to think it would in a wholesale manner have cast off its old medical friends for touts and blacklegs imported into the districts? Some of us, and not without good reason, thought we knew our public better than that.—I am, Sir, yours faithfully, F. L. Nicholls, L.R.C.S.

Fulbourn, Cambridge, April 25, 1914.

